# Synchrony and neural coding in cerebellar circuits

**DOI:** 10.3389/fncir.2012.00097

**Published:** 2012-12-11

**Authors:** Abigail L. Person, Indira M. Raman

**Affiliations:** ^1^Department of Physiology and Biophysics, University of Colorado School of MedicineAurora, CO, USA; ^2^Department of Neurobiology, Northwestern UniversityEvanston, IL, USA

**Keywords:** Purkinje, cerebellar nuclei, interpositus, corticonuclear, action potential, inhibition, IPSC, spatiotemporal

## Abstract

The cerebellum regulates complex movements and is also implicated in cognitive tasks, and cerebellar dysfunction is consequently associated not only with movement disorders, but also with conditions like autism and dyslexia. How information is encoded by specific cerebellar firing patterns remains debated, however. A central question is how the cerebellar cortex transmits its integrated output to the cerebellar nuclei via GABAergic synapses from Purkinje neurons. Possible answers come from accumulating evidence that subsets of Purkinje cells synchronize their firing during behaviors that require the cerebellum. Consistent with models predicting that coherent activity of inhibitory networks has the capacity to dictate firing patterns of target neurons, recent experimental work supports the idea that inhibitory synchrony may regulate the response of cerebellar nuclear cells to Purkinje inputs, owing to the interplay between unusually fast inhibitory synaptic responses and high rates of intrinsic activity. Data from multiple laboratories lead to a working hypothesis that synchronous inhibitory input from Purkinje cells can set the timing and rate of action potentials produced by cerebellar nuclear cells, thereby relaying information out of the cerebellum. If so, then changing spatiotemporal patterns of Purkinje activity would allow different subsets of inhibitory neurons to control cerebellar output at different times. Here we explore the evidence for and against the idea that a synchrony code defines, at least in part, the input–output function between the cerebellar cortex and nuclei. We consider the literature on the existence of simple spike synchrony, convergence of Purkinje neurons onto nuclear neurons, and intrinsic properties of nuclear neurons that contribute to responses to inhibition. Finally, we discuss factors that may disrupt or modulate a synchrony code and describe the potential contributions of inhibitory synchrony to other motor circuits.

Purkinje neurons are the principal neurons of the cerebellar cortex (Figure 1A). They receive processed sensory information via the mossy fiber to granule cell pathway as well as input from climbing fibers of the inferior olive, often considered an error or teaching signal (Marr, 1969; Albus, 1971; Gilbert and Thach, 1977; Medina et al., 2002). In addition to these excitatory synapses, Purkinje cells are subject to inhibition by basket and stellate cells. All these synaptic inputs modulate the high intrinsic firing rates of Purkinje cells (Thach, 1968; Latham and Paul, 1971). Non-vestibular Purkinje cell output is transmitted exclusively to the cerebellar nuclei, a heterogeneous set of neurons that project widely to premotor areas, as well as back to the inferior olive. The corticonuclear projection is inhibitory (Ito and Yoshida, 1966; Ito et al., 1970), and the target cells in the nuclei are also intrinsically active (Thach, 1968), setting up the specialized situation of spontaneously firing principal cells connected by inhibitory synapses.

**Figure 1:**
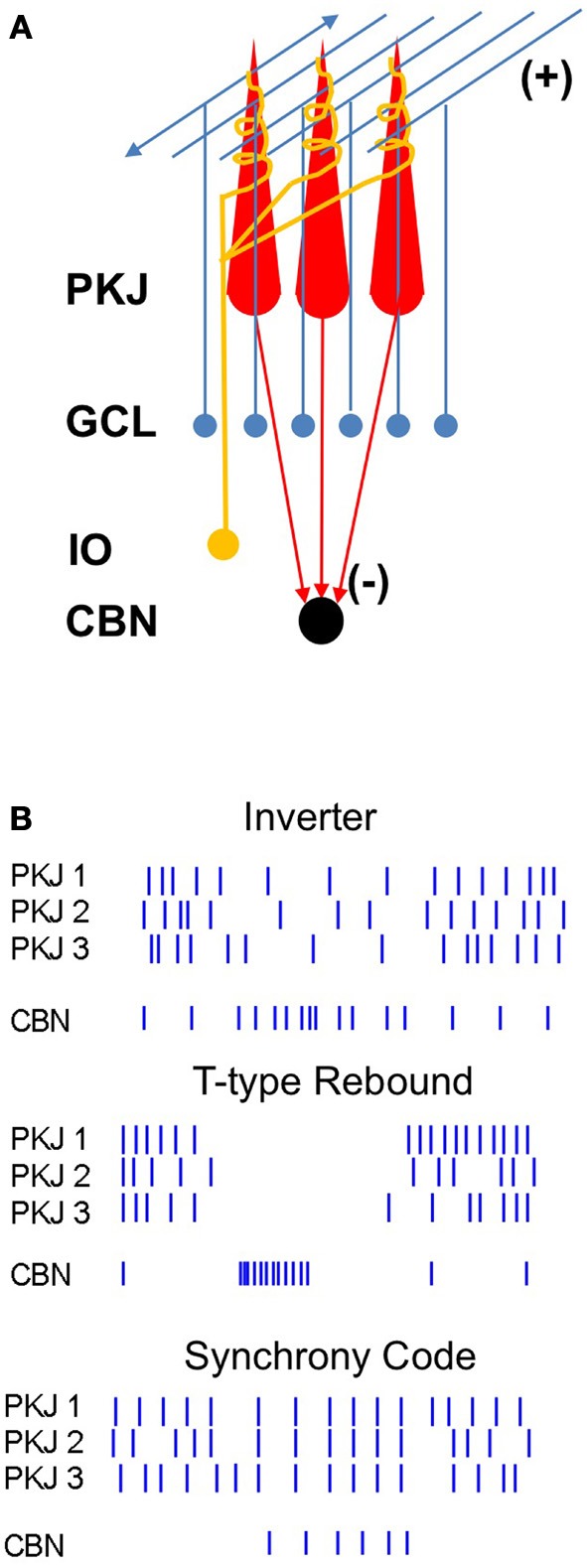
**Diagram of the corticonuclear circuit and mechanisms of corticonuclear signaling. (A)** Cerebellar Purkinje cells (PKJ) receive glutamatergic inputs from the granule cell layer (GCL) whose axons form ascending inputs onto Purkinje cells and also ramify to form the parallel fibers, as well as from the inferior olive (IO), whose axons form the climbing fibers. Molecular layer inhibitory neurons are not shown. Purkinje cells form GABAergic synapses onto neurons of the cerebellar nuclei (CBN). **(B)** Schematized spike rasters showing the key features of three non-mutually-exclusive models of Purkinje cell regulation of nuclear cell firing. Nuclear cell spikes reflect responses to three afferent Purkinje cells. *Inverter*, nuclear cell firing rate varies inversely with Purkinje cell firing rate. *T-type Rebound*, nuclear cells are largely silenced by Purkinje cell activity, but fire bursts of action potentials driven by low-voltage-activated Ca currents when Purkinje cells stop firing. *Synchrony code*, nuclear cells are silenced by asynchronous inhibition but produce short-latency spikes after IPSPs from synchronous inputs.

## Real-time coding by corticonuclear synapses

A primary question in cerebellar physiology, therefore, is how cerebellar nuclear cells transduce input from Purkinje cells to generate cerebellar output (Figure 1B). Because Purkinje neurons are GABAergic and outnumber neurons in the cerebellar nuclei—by 26:1 in the cat (Palkovits et al., 1977) and 11:1 in the mouse (Caddy and Biscoe, 1979; Harvey and Napper, 1991; Heckroth, 1994)—they are expected to exert a powerful inhibitory influence on their targets. Indeed, the fact that Purkinje cells regulate cerebellar nuclear cell output is unambiguous. Alterations of Purkinje cell firing are often associated with disease states: In motor disorders like ataxia and dystonia, changes in Purkinje action potential firing have been directly recorded in rodent models. Ataxia can result from degeneration of Purkinje cells or reductions in Purkinje spike rates (Mullen et al., 1976; Levin et al., 2006); this drastic loss of inhibitory input is expected to elevate nuclear cell firing rates. Irregular Purkinje cell firing, however, also correlates with ataxia (Walter et al., 2006). Likewise in dystonia, both Purkinje and nuclear cells fire irregular bursts of spikes (LeDoux et al., 1998). Remarkably, removing all cerebellar output by cerebellar ablation relieves dystonia, but this extreme manipulation also generates ataxia (LeDoux et al., 1993, 1998; Calderon et al., 2011). Thus, increases, decreases, and irregularities in cerebellar output all induce motor dysfunctions, indicating that both the rate and timing of spiking by cerebellar nuclear neurons must be precisely regulated under normal conditions. This regulation must be accomplished at least in part by Purkinje cells.

**Figure 2:**
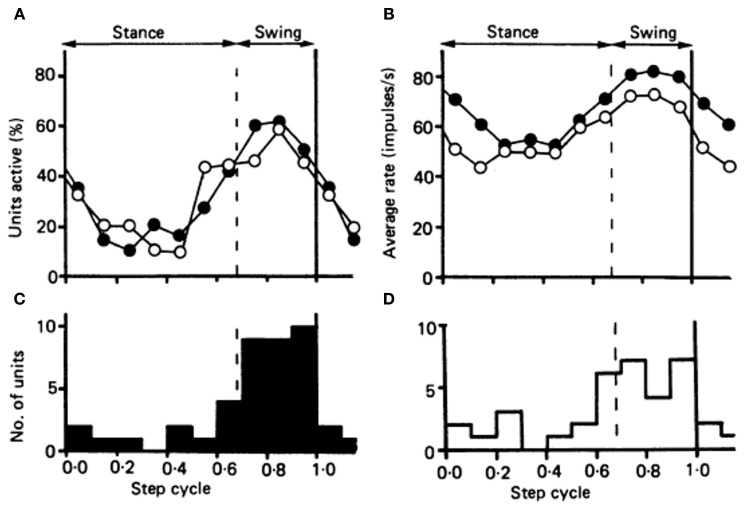
**Comparison of the timing of the activity in the population of 34 Purkinje cells in the cat identified as belonging to cl zone, with the timing of the activity in a population of forelimb-related neurons of nucleus interpositus. (A)** Plots showing the proportion of neurons in each population “active” during each tenth of the step cycle. Open circles, cl Purkinje cell population; filled circles, the population of interpositus neurons. **(B)** The fluctuation in discharge rate amongst the two populations during the course of the step cycle. Open circles represent the Purkinje cells of the cl zone; and filled circles the interpositus neurons. **(C** and **D)** Histograms showing the number of neurons attaining their peak discharge rate during each tenth of the step cycle, for the interpositus neurons and the Purkinje cells of the cl zone respectively. Reprinted from Armstrong and Edgley (1984b), with permission.

The most straightforward prediction is that firing rates of nuclear cells should be the inverse of those in their Purkinje afferents (Figure 1B, *top*). Consistent with this idea, nuclear cells often respond to sensory inputs with reduced spike rates (Armstrong et al., 1975; Cody et al., 1981; Rowland and Jaeger, 2005), suggestive of a suppression of nuclear cell activity by stimuli expected to raise Purkinje cell spike rates. Studies of populations of Purkinje neurons and cerebellar nuclear neurons, however, often show correlated changes in addition to anti-correlated changes in firing rates, both in the basal state and during behaviors associated with modulation of Purkinje cell activity. For example, in rhesus monkeys, Purkinje neurons as well as nuclear neurons increase their firing rates during brief, cue-initiated movements (Thach, 1970a,b). Similarly, during locomotion in cats, the majority of both Purkinje and interpositus neurons increase their firing rates more during forelimb swing than during stances (Figure 2; Armstrong and Edgley, 1984a,b). The absence of clearly opposing firing rate changes in these studies might be accounted for if the specific Purkinje cells and nuclear cells whose activity was recorded were not synaptically linked, but similar results have been obtained from simultaneous recordings from putative connected pairs of Purkinje and nuclear neurons. In decerebrate cats, for example, spontaneous activity of such Purkinje-nuclear pairs is not correlated, and firing rate modulation in response to periodic sensory stimuli is not consistently reciprocal, leading to the conclusion that single Purkinje afferents are insufficient to regulate the spiking behavior of nuclear cell targets (McDevitt et al., 1987). The characterization of corticonuclear synapses exclusively as inverters, therefore, may be an oversimplification.

The alternative to nuclear cells' tracking the spike rate of individual Purkinje afferents is that it is the activity of populations of Purkinje cells that encodes meaningful signals. This idea has been supported by studies of the oculomotor system of rhesus monkeys. In a task involving visually guided saccades, bursts produced by individual Purkinje cells turn out to be imperfect predictors of saccade duration, but saccade onset and termination is precisely represented by the activity of a population of Purkinje cells considered together (Thier et al., 2000; Catz et al., 2008). The authors point out that the idea that neurons of the cerebellar nuclei respond to groups of Purkinje cells rather than to individual cells is virtually an obligate outcome of the anatomical convergence of Purkinje cells onto nuclear cells.

Nevertheless, when the question is further examined at the cellular level, paradoxes remain. Both Purkinje neurons and their target cells in the nuclei spontaneously fire tens of spikes per second in the absence of synaptic input (Llinás and Sugimori, 1980; Jahnsen, 1986a; Mouginot and Gähwiler, 1995; Häusser and Clark, 1997; Raman and Bean, 1997). The high basal firing rates of Purkinje cells (>50 spikes/s), extensive inhibitory innervation of nuclear cell somata and proximal dendrites (Chan-Palay, 1977; Palkovits et al., 1977; De Zeeuw et al., 1994; Sugihara et al., 2009), and minimal synaptic depression (Mouginot and Gähwiler, 1995; Telgkamp et al., 2004) predict a complete shunt of nuclear cells even in the basal state. Nuclear cells, however, show basal firing rates of >20 spikes/s in the brains of monkeys, cats, rats, and mice (Thach, 1968; Rowland and Jaeger, 2005; Bengtsson et al., 2011; Blenkinsop and Lang, 2011; Person and Raman, 2012), raising the question of what types of signals are required to suppress—or trigger—nuclear cell firing. One possibility is that nuclear cells are indeed fully silenced by Purkinje inhibition, so that their spiking relies entirely on excitation by mossy fibers, an idea that emerged from models based on early recordings of synaptic properties (Anchisi et al., 2001; Gauck and Jaeger, 2003). This idea, however, implies that the intrinsic activity of nuclear cells is necessarily suppressed by the intrinsic activity of Purkinje cells, a surprisingly energetically costly mode of re-establishing the common scenario of a silent principal cell that requires excitation to fire. Another possibility is that only when Purkinje cells cease firing for prolonged periods, on the order of a few hundred milliseconds, do nuclear cells fire post-inhibitory “rebound” spikes (Llinás and Mühlethaler, 1988; Figure 1B, *middle)*. This idea is attractive, especially given the high densities of low-voltage activated “T-type” Ca currents in nuclear cells (Aizenman and Linden, 1999; Czubayko et al., 2001; Molineux et al., 2006). The notion that nuclear cells generate action potentials only when afferent Purkinje cells are collectively silenced for durations long enough to permit recovery of T-type Ca channels, however, implies that information encoded in spike rates of Purkinje cells may not be transmitted. Moreover, it predicts lags between Purkinje and nuclear cell activity, inconsistent with cerebellar response latencies of a few milliseconds (Mauk and Buonomano, 2004).

## Synchronous firing by purkinje cells

A resolution to these paradoxes may emerge from the observation that populations of Purkinje cells can synchronize their spiking, especially during cerebellar behaviors. It has long been recognized that multiple Purkinje cells coincidently fire complex spikes (Sasaki et al., 1989), a synchrony that may result from innervation of several Purkinje cells by a common climbing fiber, but which is likely intensified by simultaneous firing by inferior olivary cells promoted by gap junctions (Lampl and Yarom, 1993; Devor and Yarom, 2002; Blenkinsop and Lang, 2006). In contrast to the inconsistent responses to Purkinje cell simple spike rate, inhibition of nuclear cells by complex spikes is more readily evident experimentally. Spontaneous complex spikes in Purkinje neurons of ketamine-xylazine anesthetized rats can elicit prolonged inhibitory responses in connected nuclear cells (Blenkinsop and Lang, 2011). Likewise, in decerebrate cats, inferior olivary stimulation elicits giant IPSPs in whole-cell recordings of cells in the cerebellar nuclei (Bengtsson et al., 2011).

Complex spikes may be particularly effective at inducing overt inhibition of nuclear cells because the multiple spikelets in each complex spike propagate as two or three high-frequency action potentials (Khaliq and Raman, 2005; Monsivais et al., 2005), likely eliciting a brief burst of postsynaptic IPSCs. An alternative, not mutually exclusive interpretation, however, is that a key parameter is the synchrony with which Purkinje cells tend to fire complex spikes (Welsh et al., 1995; Mukamel et al., 2009; Ozden et al., 2009; Schultz et al., 2009). If some of these coincidently firing Purkinje cells converge, nuclear cells may be subject to inhibition from many Purkinje cells at once. By extension, the termination of the synchronous IPSP may provide a window in which spike probability is transiently increased owing to the relief of inhibition.

The temporal relationship of action potential firing by multiple convergent Purkinje cells may therefore be a central factor in determining nuclear cell responses to inhibitory signals (see also De Zeeuw et al., 2011). This idea is particularly interesting because Purkinje cells tend not only to generate complex spikes coincidently, but also to fire simple spikes synchronously during both movement and sensory stimulation. In one of the earliest demonstrations of simple spike synchrony, Bell and Grimm (1969) made double microelectrode recordings in pentobarbital-anesthetized cats and showed that Purkinje neurons located close together (<70 μm) often fired nearly simultaneously. Cross correlograms, calculated with 1-ms resolution, consistently showed peaks at 0 delays. Similar observations were made in pentobarbital-anesthetized guinea pigs, in which Purkinje neurons located along parallel fiber beams showed large cross-correlation peaks at 1 ms (Bell and Kawasaki, 1972). Since these early studies, numerous groups have observed synchronous simple spikes, in several preparations, with several anesthetic regimens. A consistent finding is that Purkinje cells that synchronize with one another are also located near one another, such that submillisecond correlations at time 0 are evident only in Purkinje neurons that are fewer than 100 microns apart (MacKay and Murphy, 1976; Ebner and Bloedel, 1981; De Zeeuw et al., 1997; Shin and De Schutter, 2006; Heck et al., 2007; de Solages et al., 2008; Bosman et al., 2010; Wise et al., 2010).

**Figure 3:**
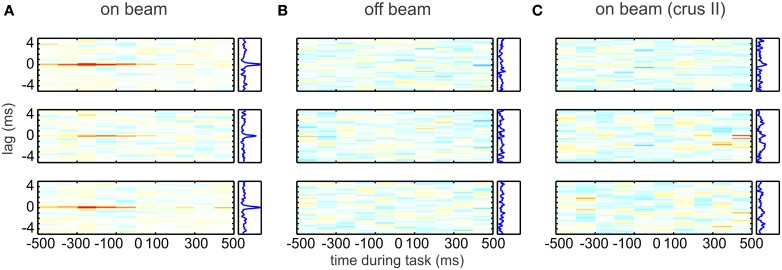
**Simple spike synchrony in Purkinje cells.** On-beam Purkinje cells (Pcs) in the paramedian lobe fired precisely synchronized simple spikes (SSs) time-locked to behavior. Each plot represents the time-resolved cross-correlogram of Pc SS activity recorded at two different electrodes during reaching–grasping movements by awake rats. Average cross-correlations were calculated for epochs of 100-ms duration with a temporal resolution of spike delays of 40 μs. In each plot, the abscissa indicates time during the movement. At time 0, the rat has completed paw extension and touches the food pellet. The ordinate indicates the cross-spike train interval. All plots use the same color map (from red = 0.01 through white = 0 to blue = −0.01). Plots to the right side of the time-resolved cross-correlation matrix show the average excess correlation, i.e., the integral along the abscissa. The time-resolved cross-correlations shown here were generated from the data shown in Figure 1 of Heck et al. (2007). Data were analyzed by subdividing the experimental time into 100-ms bins and calculating the average excess correlation within each bin at 40-μ s resolution. The resulting color-coded matrix was smoothed across delays by convolving with a 120-μs Gaussian. **(A)** Correlation analysis of spike activity recorded with the electrode array in on-beam orientation in the paramedian lobe. (Top to Bottom) Plotted cross-correlations of spike activity recorded on electrode 1 vs. 2, 2 vs. 3, and 1 vs. 3. Behavior-related occurrence of on-beam synchronous activity is visualized by the red lines at zero lag in the on-beam matrix. The occurrence of synchronous activity was not directly correlated with spike rate or change of rate. **(B)** (Top to Bottom) Arrangement of cross-correlation plots as in **(A)**. Here, the electrode array was in off-beam orientation. No synchronous activity was seen in paired off-beam recordings. **(C)** Correlation analysis of SS activity recorded during behavior in crus II, an area outside the arm representation, with the electrode array in on-beam orientation. No behavior-modulated SS activity or synchronous firing was observed. Reprinted from Heck et al. (2007), with permission.

Synchrony is subject to modulation by sensory input or motor behaviors associated with cerebellar activity. Several groups report that synchrony is enhanced by somatosensory or proprioceptive stimulation (MacKay and Murphy, 1976; Ebner and Bloedel, 1981; Wise et al., 2010). In a breakthrough series of experiments, Heck et al. (2007) demonstrated that bands of Purkinje neurons fired synchronously during a learned motor task in rats, and that the increase of synchrony was time-locked to movement (Figure 3). Not only was synchrony resolved with high temporal precision, in cross correlations with bins <1 ms wide, but it was also shown to be widespread, with synchrony being evident in most Purkinje pairs located within a few hundred microns of each other.

## Mechanisms of simple spike synchrony

Despite the repeated and robust observations of Purkinje cell simple spike synchrony, results vary regarding *which* Purkinje cells synchronize and *how* they do so. Some studies report simple spike synchrony in the parasagittal plane within microzones of Purkinje cells that fire synchronous complex spikes; in these studies, cells with synchronous complex spikes were more likely also to fire simple spikes synchronously, suggestive of a common mechanism underlying both types of action potentials (Bell and Kawasaki, 1972; Wise et al., 2010). In contrast, in several preparations, synchrony is evident in on-beam Purkinje cells, expected to be innervated by common parallel fibers, but different climbing fibers (MacKay and Murphy, 1976; Ebner and Bloedel, 1981; Heck et al., 2007; Bosman et al., 2010). Interestingly, some of this work reporting on-beam synchrony was initially directed toward documenting the functional correlate of delay lines predicted by parallel fiber anatomy (Braitenberg, 1967; Eccles et al., 1967), and sought to find sequential, not synchronous Purkinje cell firing. Even examining Purkinje responses separated by a range of distances (0.1–1.5 mm in anesthetized rats), however, reveals no such delay-line like behavior (Jaeger, 2003). These experiments add to the growing body of data demonstrating that, despite their striking anatomy, parallel fibers may not effectively deliver information to Purkinje cells in a precise temporal sequence (e.g., Bower and Woolston, 1983).

Granule cells may, nevertheless, play a significant role in synchronizing simple spikes. One proposal is that Purkinje simple spike synchrony arises from common input patterns from ascending granule cell axons that receive inputs from the same mossy fibers (Heck et al., 2007). In this case, small groups or “patches” of granule cells would synchronize simple spikes in Purkinje cells in a restricted transverse and parasagittal region. Consistent with this idea, in response to muscle stretch, the shortest response latencies among granule cells are of those that lie directly beneath the responsive region of Purkinje neurons (MacKay and Murphy, 1976; Murphy et al., 1976). In addition, direct mossy fiber stimulation only activates groups of Purkinje cells located directly above the mossy fiber termination zone (Eccles et al., 1972; Bower and Woolston, 1983; Cohen and Yarom, 1998; Bower, 2002; Dizon and Khodakhah, 2011).

Both anatomical and physiological studies have led to the proposal that ascending axons may have specializations that permit them to drive Purkinje spiking more effectively than do parallel fibers. Electron microscopic studies reveal larger synaptic volume and more vesicles in ascending than in parallel fibers (Gundappa-Sulur et al., 1999). Electrophysiological recordings in slices report that more ascending synapses than parallel fiber synapses are functional (Isope and Barbour, 2002) and that ascending branches have higher release probability and are relatively resistant to long-term depression (Sims and Hartell, 2005, 2006). These attributes, however, do not indicate that ascending axons necessarily evoke larger unitary responses in Purkinje cells than parallel fibers do, and, in fact, evidence that both ascending and parallel fiber inputs are functionally equivalent has also been presented (Isope and Barbour, 2002; Walter et al., 2009; Zhang and Linden, 2012).

Regardless of the relative strength of the two branches of granule cell input, Purkinje neurons may still be preferentially excited by granule neurons directly beneath them. In rat cerebellar slices, stimulating granule cells leads to excitation of the Purkinje cell located just superficially, while stimulating more lateral groups of granule cells (in the parasagittal plane) recruit inhibitory inputs to that Purkinje cell, by activating molecular layer interneurons (Dizon and Khodakhah, 2011). These data suggest that simple spike synchrony would require co-activation of several discrete groups of granule cells to overcome lateral inhibition by basket and/or stellate cells. In addition, the inhibition of Purkinje cells by basket and stellate cells (in coronal slices, along the parallel fibers) has been reported to provide a feedforward inhibition that narrows the duration of granule-cell mediated excitation to 1–2 ms (at 32–35°C), an effect that would be expected to increase the precision of Purkinje cell firing in response to excitation (Brunel et al., 2004; Mittmann et al., 2005; Kanichay and Silver, 2008; D'Angelo and De Zeeuw, 2009; Dizon and Khodakhah, 2011). Consistent with this idea, Shin and De Schutter (2006) found that simple spikes separated by longer intervals (>12 ms) were more likely to synchronize than those with shorter gaps, such that the onset or offset of longer pauses were likely to occur simultaneously. This synchrony of pauses was not attributable to complex spikes, and thus seems likely to involve local inhibition.

Other forms of inhibition may actively facilitate simple spike synchrony. For instance, in a clever series of pharmacological experiments, de Solages et al. (2008) demonstrated that blocking GABA_A_ receptor-mediated inhibition disrupted synchrony; suppressing activity of inhibitory interneurons with cannabinoid (CB1) receptor agonists, however, left synchrony unaffected, leading to the conclusion that Purkinje–Purkinje inhibition, mediated by local collaterals, provided the GABAergic signals that were crucial to maintaining synchrony. This idea is consistent with computational work demonstrating that synchrony is an extremely common consequence of synaptically connected oscillatory inhibitory cells (Salinas and Sejnowski, 2001). Coupling by gap junctions between Purkinje neurons and molecular layer interneurons has also been proposed to support synchronization of Purkinje cells (Middleton et al., 2008).

An alternative mechanism for simple spike synchrony involves complex spikes organizing simple spikes. As mentioned above, complex spikes occur synchronously in Purkinje neurons across parasagittal bands of the cerebellar cortex. Since the cell bodies of climbing fibers, which drive complex spikes in Purkinje cells, are electrically coupled in the inferior olive (Llinás and Yarom, 1986; Lampl and Yarom, 1993; Devor and Yarom, 2002; Blenkinsop and Lang, 2006) and since single climbing fibers can contact multiple Purkinje neurons, coincident activity in multiple climbing fibers tends to drive a sizable population of Purkinje neurons to fire synchronous complex spikes (Welsh et al., 1995; Mukamel et al., 2009; Ozden et al., 2009; Schultz et al., 2009), leading to the measurable inhibition of nuclear neurons described above. Of possible significance for simple spike synchrony, complex spikes are often followed by a pause in firing. If several Purkinje cells with common basal firing rates were to experience pauses of equivalent durations, the synchronous complex spikes might lead to a phase resetting of simple spikes, such that simple spikes in a population of Purkinje neurons would be synchronized upon resumption of firing. Although this scenario presents an intriguing potential link between complex and simple spike synchrony, pauses following complex spikes tend to be highly variable, ranging from tens to hundreds of milliseconds even within cells (Bell and Grimm, 1969; Latham and Paul, 1971; Murphy and Sabah, 1971; McDevitt et al., 1982; Steuber et al., 2007), suggesting that additional factors may be necessary to achieve a precise phase resetting by complex spikes. The generation of simple spike synchrony may thus rely on several mechanisms working together—or different mechanisms active under different conditions. Nevertheless, the observation that synchrony increases during behaviors that involve the cerebellum suggests that synchronously firing Purkinje neurons may encode information that is specifically transmitted to the cerebellar nuclei.

## Purkinje-to-nuclear convergence: 1000s, 100s, or 10s?

Understanding the extent to which synchronous and asynchronous Purkinje inhibition differentially affect nuclear cells requires answering the apparently basic question of how many Purkinje cells converge onto a cerebellar nuclear neuron. This information will define the basal level of inhibition, which in turn will influence how many Purkinje cells must synchronize to be detected by the nuclear cell. The most widely cited estimate of the convergence of Purkinje neurons onto nuclear neurons comes from the heroic and careful quantitative electron microscopic study of the cat by Palkovits et al. (1977). Comparing the total number of “synaptic profiles,” or boutons, of Purkinje cells to the total number of neurons in the cerebellar nuclei led to an estimate of 11,600 Purkinje boutons per nuclear cell, with each Purkinje cell calculated to have 474 boutons. The number of nuclear cells targeted by each Purkinje cell, i.e., the degree of divergence, was estimated to be roughly 35 from the number of nuclear cells that could fall within the axonal arbor of the Purkinje cell, a number that Palkovits et al. described as an “order of magnitude” measurement. Thus, each nuclear cell was calculated to receive 474/35 or 13.5 boutons from any given Purkinje cell. Dividing 11,600 by 13.5 gave the final value of convergence of 860 Purkinje neurons per nuclear cell.

As the authors acknowledged, the degree of divergence was only weakly constrained, making the number of boutons from each Purkinje neuron synapsing onto each nuclear cell also only loosely approximated. An updated measure of the number of boutons from a single Purkinje cell contacting an individual nuclear cell can be obtained from physiological measurements, however. In brain slices from mice, the ratio of the unitary IPSC to the miniature IPSC estimates the quantal content at 12–18 (Telgkamp and Raman, 2002; Pedroarena and Schwarz, 2003; Person and Raman, 2012) and the release probability per bouton is near 0.5 (Telgkamp et al., 2004). Multiplying these values gives an estimate of the number of boutons at a single Purkinje-nuclear contact at 24–36; this higher value is within the range (1–50) proposed by Palkovits et al. for the cat, and is therefore unlikely to reflect species differences. Incorporating this value into their calculation reduces the estimate of convergence to 322–483.

Perhaps more importantly, as the authors state explicitly, their “apparently excessive” numerical estimate for average convergence is based on the simplifying assumption that each Purkinje cell ramifies to the same extent on all nuclear cells. They point out, however, that Purkinje neurons consistently make non-uniform contacts onto nuclear cells, occasionally “erupting” into “numerous (~50) boutons all in contact with the same cell body.” Ramón y Cajal similarly observed that each Purkinje neuron axon formed six to eight “nests” onto as many cells (Chan-Palay, 1977). Such dense terminal perisomatic plexes are also mentioned in other descriptions of Purkinje axonal arbors (Chan-Palay, 1977; Bishop et al., 1979; De Zeeuw et al., 1994; Wylie et al., 1994; Teune et al., 1998; Sugihara et al., 2009), suggesting that they are a common specialization of Purkinje neuron terminals. Palkovits et al. (1977) propose that each Purkinje neuron may have 3–6 primary targets, while providing weak input to many more, and conversely that nuclear cells may only receive strong somatic input from “several”—that is, a relatively small number of— Purkinje cells (see also Sugihara et al., 2009).

The scenario of a few dozen dominant Purkinje cell inputs per nuclear cell is consistent with physiological measurements. From recordings either in Deiter's nucleus *in vivo* or in the cerebellar nuclei in an acute brain slice preparation, the ratio of the maximal and minimal responses evoked by Purkinje cell activation gives convergence estimates of 22–30 *in vivo* (Eccles et al., 1967) and 10–20 *in vitro*; the latter estimate provides only a lower bound, given that some inputs are likely lost during slicing (Person and Raman, 2012). Additional measurements, however, constrain the functional convergence in the mouse to be between 30 and 50. First, based on anatomical measurements of cell surface area, boutonal area, and extent of inhibitory innervation (Chan-Palay, 1977; Telgkamp et al., 2004; Uusisaari et al., 2007), it seems likely that a large nuclear neuron can likely maximally accommodate 1250 Purkinje boutons. A similar calculation has recently been made for the cat, estimating the number of inhibitory synapses at 600–1200 (Bengtsson et al., 2011). (Note that this value is an order of magnitude lower than the estimate of 11,600 of Palkovits et al. (1977). Person and Raman (2012) initially proposed that the high estimate might have resulted from counting synaptic densities and assuming one rather than ~10 densities per bouton, but this does not seem to be the case). Dividing 1250 boutons on each nuclear cells by 24–36 boutons per Purkinje-nuclear contact (as described above) predicts 34–52 Purkinje cells/nuclear cell (Person and Raman, 2012). Corroborating this result, dividing the maximal GABA_A_-evoked conductance in a nuclear cell by the unitary IPSC predicts that a maximum of 30 Purkinje cells, evoking currents of the mean unitary size, converge onto the nuclear cell (Person and Raman, 2012).

Interestingly, by analyzing single reconstructed Purkinje axons at the light microscope level, Sugihara et al. (2009) quantified the number of putative axonal boutons (axonal swellings) made by individual Purkinje axons in rats, reporting values between 120 and 150 (somewhat lower than the 474 originally estimated in the cat). If each Purkinje axon contributes ~30 boutons per nuclear neuron, then divergence must be 4–5, values that align well with numerical ratios of Purkinje to nuclear neurons of 11:1 in the mouse and convergence of ~50:1.

## Do convergent purkinje cells synchronize?

**Figure 4:**
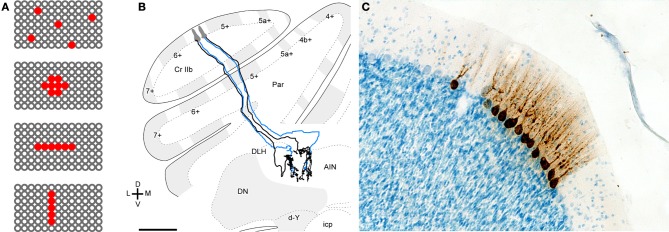
**Evidence for related functional roles of neighboring Purkinje cells. (A)** Schematic illustrating a surface view of the cerebellar cortex with red indicating possible patterns of convergent Purkinje cells. Converging Purkinje cells can be (top to bottom) widespread, clustered, on-beam, or ordered parasagittally. **(B)** Projections of two rat Purkinje cells that were separated transversely but located in the same aldolase C compartment. Two small injections were made in medial and lateral 5- in the apex of crus IIa. Purkinje cell axons that originated from each of the injections were reconstructed. Blue axons indicate those that were partially reconstructed, except for the fine branches in the terminal arbor. The medial Purkinje cells projected to the lateral anterior interpositus nucleus (AIN) while the lateral Purkinje cells projected to the junction between the dorsolateral hump (DLH) of the anterior interpositus and lateral anterior interpositus nucleus. Scale bar = 500 μm. Panel **(B)** reprinted from Sugihara et al. (2009), with permission. **(C)** Labeling of Purkinje after injection of rabies virus into various limb muscles of the rat. Microphotographs showing labeling of a cluster of Purkinje cells in the lateral vermis. Purkinje cells within such a cluster display a uniform level of infection. Scale bar: 100 μm. Panel **(C)** reprinted from Ruigrok et al. (2008), with permission.

For inhibitory synchrony to influence firing by nuclear neurons, synchronously firing Purkinje cells must converge on a common target neuron. Numerous studies have established that the olivocerebellar loops are highly topographically organized, suggesting that neighboring Purkinje neurons target neighboring nuclear neurons (Figures 4A,B; Groenewegen and Voogd, 1977; Andersson and Oscarsson, 1978; Buisseret-Delmas and Angaut, 1993; Sugihara et al., 2009). Since synchrony of simple spikes appears restricted to neighboring Purkinje neurons, it seems likely that target nuclear neurons indeed receive synchronous inhibitory input. Unfortunately, it remains technically unfeasible to record simultaneously from a population of Purkinje neurons with known convergence and their target neurons. Advances in transneuronal labeling and imaging will be necessary before such experiments become possible. Nevertheless, restricted tract tracing of neighboring Purkinje neurons reveal intermingled terminal arbors in the nuclei (De Zeeuw et al., 1994; Sugihara et al., 2009). Furthermore, tantalizing data from transsynaptic viral tracing from muscle labels small patches of 3–10 directly adjacent Purkinje neurons (Figure 4C; Morcuende et al., 2002; Ruigrok et al., 2008; Sun, 2012). It seems likely that these patches not only form functional units related to a given muscle, but also may indeed converge on a common target neuron.

## Corticonuclear signaling: what purkinje neurons can tell nuclear neurons

On the assumption that synchronously firing Purkinje cells indeed converge on target neurons, the question becomes how nuclear cells transduce this synchrony. While the idea of rebound bursts in response to the concerted offset of prolonged inhibition has been discussed for decades (Jahnsen, 1986b; Llinás and Mühlethaler, 1988), the idea that the relief of synchronous inhibition permits simple spiking by nuclear cells was initially proposed by Gauck and Jaeger (2000). Using dynamic clamp to simulate excitatory and inhibitory inputs to nuclear cells in rat cerebellar slices, they demonstrated that nuclear cell simple spike probability increased whenever simulated Purkinje cell inhibition was reduced. As the synchrony of Purkinje input increased, so did the reliability that a spike would occur after coincident IPSPs, raising the overall nuclear cell spike rate (Figure 1B *bottom*; see also Hoebeek et al., 2010, below). Later modeling studies further predicted that if the simulated convergence ratio of Purkinje cells onto nuclear cells were decreased, a modification comparable to an increase in synchrony, nuclear cells would tend to spike more in the synchronous gaps in inhibition (Luthman et al., 2011). The insight that the degree of inhibitory synchrony is a key determinant of cerebellar output may be central to resolving the paradoxes in Purkinje-to-nuclear cell signaling described above.

The quantitative components of the model by Gauck and Jaeger, however, were limited by the experimental data available at the time. The first recordings of Purkinje mediated IPSCs were obtained from young rats at room temperature (Anchisi et al., 2001), which estimated the decay time constant of IPSCs to be near 14 ms, and convergence was set at 860 based on the work of Palkovits et al. (1977). As a consequence, nuclear cells were shunted by ongoing inhibition. Related results came from cerebellar slices from ~2 week old mice, in which IPSPs evoked by stimulating multiple Purkinje afferents at 50–100 Hz at 31°C led to standing or tonic inhibitory current that tended to shunt nuclear cell spiking. In contrast, IPSPs evoked at 10 Hz delayed the spikes of cerebellar nuclear cells spontaneously firing between 15 and 25 Hz, such that nuclear cells spikes entrained to the 10-Hz input (Telgkamp and Raman, 2002). While this observation provided an interesting example of how gaps in Purkinje mediated inhibition could permit nuclear cell spikes to escape, the observation was hard to relate to anything physiological, since such low frequency firing does not usually typify Purkinje cells.

**Figure 5:**
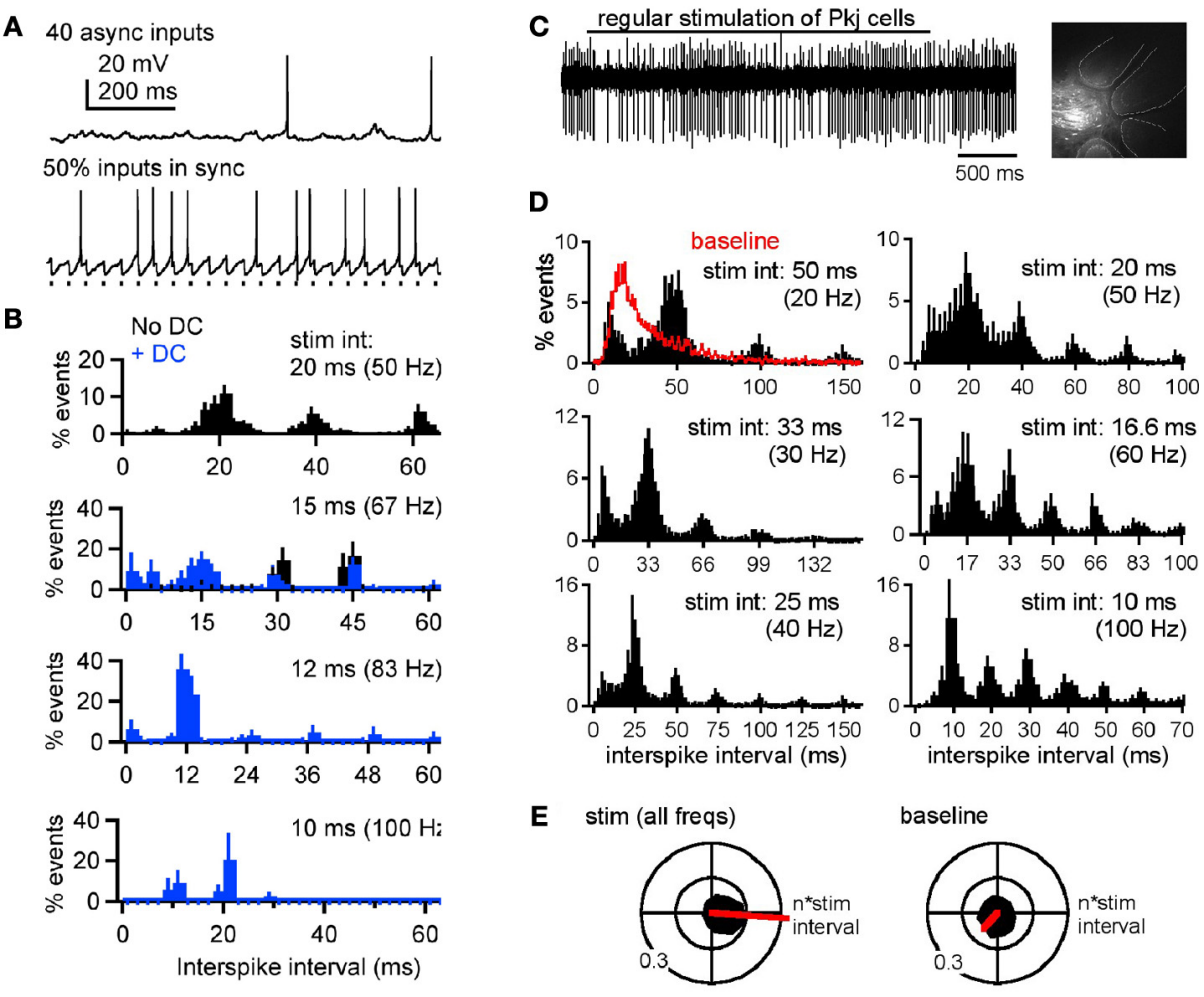
**Synchronous Purkinje inputs set spike timing of nuclear neurons, *in vitro* and *in vivo*. (A)** Responses of a whole-cell current-clamped cerebellar nuclear neuron in a mouse cerebellar slice to dynamically clamped (dyn) IPSPs mimicking 40 asynchronous (top) or 20 asynchronous and 20 synchronous Purkinje inputs (bottom). **(B)** Normalized *interspike* interval distributions during partially synchronized (50%, i.e., 20 out of 40) dynIPSPs, where the rates of the synchronous input ranged from 50 to 100 Hz. Abscissa tick marks indicate multiples of the *interstimulus* intervals of the synchronous subpopulation. Bin width, 2 ms. Black: no current injection. Blue: with 200 pA steady current (DC) applied to increase spike probability during inhibition. **(C)** Responses of an extracellularly recorded cerebellar nuclear neuron in a ketamine-xylazine anesthetized mouse. Upper trace, response of a nuclear neuron to 40-Hz molecular layer stimulation (bar). Inset, recording site in the cerebellar nuclei recovered after focal Alexa 568 injection. Dashed lines demarcate cerebellar folia. Scale bar, 200 μm. **(D)** Mean normalized interspike interval distributions during molecular layer stimulation from 20 to 100 Hz. Abscissa tick marks indicate multiples of the interstimulus intervals. Red baseline histogram includes intervals before and after stimulation. Bin width, 2 ms. **(E)** Black: polar histograms of interspike intervals during stimulation across rates (left) or during baseline periods. Red: net vectors of polar histograms. Reprinted from Person and Raman (2012), with permission.

Our recent work (Person and Raman, 2012), however, illustrates two additional features of cerebellar nuclear cells that are likely to exert a significant influence on the response to simple spike synchrony under physiological conditions. First, in cerebellar slices of weanling mice, recorded at near-physiological temperatures (36–37°C), the intrinsic firing rates of cerebellar nuclear cells are near 90 spikes/s, a value that is considerably higher than the 20 spikes/s recorded in younger (~2 weeks old) animals that have been the focus of most earlier studies (Aizenman and Linden, 1999; Czubayko et al., 2001; Telgkamp and Raman, 2002). Second, the IPSC kinetics decay with a time constant of about 2.5 ms, a time course that is much briefer than at the cooler temperatures at which the majority of previous recordings have been made (Anchisi et al., 2001; Telgkamp and Raman, 2002; Pedroarena and Schwarz, 2003; see also Uusisaari and Knöpfel, 2008). As a consequence of their very brief IPSCs and fast intrinsic firing, nuclear cells generate short-latency, well-timed action potentials immediately after synchronous IPSPs and can entrain to synchronous inhibition at much higher frequencies. Even when only a subset of afferents synchronizes—as few as 2 out of 40—the spike probability increases immediately after the synchronized IPSPs. As a result, when Purkinje cells are made to fire synchronously at a regular frequency, the interspike interval histograms of nuclear cell spikes show peaks at multiples of the interval between synchronous IPSPs (the interstimulus interval). Thus, elements of the spike timing of synchronously firing Purkinje cells can be encoded in nuclear cell output. This phenomenon is evident *in vitro*, with dynamically clamped IPSC inputs, as well as *in vivo* in ketamine-xylazine anesthetized mice, with electrical stimulation of the molecular layer applied to synchronize Purkinje cell firing (Figure 5; Person and Raman, 2012). If such a phenomenon persists in alert animals, then synchronized inhibitory input may dictate the output of cerebellar nuclear cells. Indeed, in awake mice, single stimuli applied to the paravermal lobes, which likely evoke synchronous action potentials in multiple Purkinje cells, elicit “timed spiking” in their nuclear cell targets, i.e., an increased probability of action potential firing at a fixed short latency (~10 ms) after the stimulus (Hoebeek et al., 2010). Assuming that this observation can be extended to a train of simple spikes, the spike timing of coincidently firing Purkinje cells may be relayed to downstream targets. As synchrony shifts from one group of Purkinje cells to another, different subsets of Purkinje cells may control cerebellar output at different times.

For nuclear cells to encode timing even of high-frequency Purkinje inputs, the kinetics of IPSCs must be highly constrained. In slice recordings made at subphysiological temperatures, IPSCs decay in 5–15 ms, and trains of IPSPs applied at frequencies at or above 50 Hz fully suppress nuclear cell firing (e.g., Aizenman and Linden, 1999; Telgkamp and Raman, 2002; Pedroarena and Schwarz, 2003). In contrast, at 36–37°C, similar stimuli do not silence nuclear neurons, instead permitting spikes to escape occasionally between IPSPs. That this persistence of firing depends on IPSC kinetics is evident from dynamic clamp studies prolonging the decay time to that measured at room temperature and 31°C (Person and Raman, 2012). Comparably fast kinetics have been reported only rarely (Bartos et al., 2001). Thus, the rapid gating of nuclear cell IPSCs emerges as a key specialization required for nuclear cells to follow high-frequency, synchronous Purkinje inputs.

It is worth emphasizing, however, that despite this control of spike timing, the net effect of Purkinje activity is not excitatory. In slices with dynamically clamped inhibitory inputs and fast synaptic excitation blocked pharmacologically, spike rate always dropped relative to the spontaneous spike rate. Importantly, however, firing rates of nuclear cells varied not only according to the spike rate of the synchronous inhibitory inputs, but also according to the number of afferents that synchronized. A greater percent synchrony favored higher firing rates, consistent with paired recordings demonstrating that the output rate of nuclear cells could not be predicted from the input rate of one afferent Purkinje cell (McDevitt et al., 1987). Likewise *in vivo*, with excitation unblocked but probably reduced owing to ketamine-xylazine anesthesia, the net firing rate of nuclear cells generally decreased with molecular layer stimulation (Person and Raman, 2012), consistent with studies showing reduced nuclear cell firing in response to sensory stimuli expected to raise the activity of Purkinje cells (Rowland and Jaeger, 2005). In fact, the proposed cellular mechanism (see below) predicts that ongoing inhibition cannot accelerate firing, but simply permits intrinsically driven action potentials to escape during transient gaps in inhibition induced by synchrony. Since these action potentials do not necessarily occur after every synchronous IPSP, the output rate of nuclear cells is likely to be lower than the input rate of coincidently firing Purkinje cells. Nevertheless, the spikes that do occur will be time-locked to the synchronous input.

It is also appropriate to stress that the idea that Purkinje simple spike synchrony is a key factor in determining nuclear cell output, does not make predictions about whether sensorimotor information transmitted out of the cerebellum is encoded either in spike rates or in spike times of nuclear cells. Instead, synchronization of Purkinje cell spiking will necessarily affect both the rate and timing of nuclear cell action potentials. Specifically, if nuclear cell action potentials follow synchronized IPSPs with highly consistent latencies, the relative timing of Purkinje spikes will be faithfully preserved. If these spikes occur with fixed probability, rate information will be encoded as well. If jitter arises, however, spike timing information from afferent Purkinje cells may be degraded, but the firing rate may be still be relayed. Indeed, a preliminary report suggests that nuclear cells integrate Purkinje firing rates over a 12.5 ms window (Cao et al., 2012); the brevity of this window (2 spikes for a Purkinje cell firing at 80 Hz) is consistent with a mild jitter on a putative response to synchronized IPSPs.

## Mechanisms of post-inhibitory action potential firing

What is the ionic basis of cerebellar nuclear cell action potentials that follow synchronized IPSPs? As mentioned above, nuclear neurons are known for their expression of low-voltage-activated or T-type Ca channels (Llinás and Mühlethaler, 1988; Muri and Knöpfel, 1994; Aizenman et al., 1998; Czubayko et al., 2001; Gauck et al., 2001; Molineux et al., 2006). Because these channels activate at relatively hyperpolarized voltages, inactivate within tens of milliseconds, and recover at strongly hyperpolarized potentials (Zheng and Raman, 2009), they are well suited to produce high-frequency bursts of action potentials after periods of strong hyperpolarization. It remains a question, however, what types of physiological stimuli maximally recruit and activate these currents. Purkinje-mediated inhibition alone is not an ideal candidate, as IPSPs cannot hyperpolarize neurons beyond E_Cl_—near −75 mV in nuclear cells—a voltage at which recovery of T-type current is minimal (Jahnsen, 1986b; Zheng and Raman, 2009). Indeed, cerebellar slices from guinea pig, post-IPSP bursts are not evident (Jahnsen, 1986b), and in cerebellar slices from mouse, little T-type current is evoked in either the somatic or dendritic compartments even after 300-ms high-frequency trains of IPSPs that silence postsynaptic firing (Zheng and Raman, 2009). Furthermore, in anesthetized rats, high-frequency stimulation of Purkinje neurons only rarely elicited rebound-like bursts of action potentials in nuclear neurons (Alviña et al., 2008). Studies in rat cerebellar slices nevertheless suggest that the small fraction of T-type current that recovers during 300-ms IPSP trains is sufficient to increase burst probability (Engbers et al., 2011); such periods of spike suppression can also lead to prolonged post-inhibitory firing through additional ionic mechanisms (Sangrey and Jaeger, 2010; see also Zheng and Raman, 2011). It is unlikely, however, that T-type currents are engaged by single synchronous IPSPs, which hyperpolarize cells no further than about −70 mV for durations only on the order of ten milliseconds (Jahnsen, 1986b; Person and Raman, 2012). Instead, action potentials that occur after synchronous IPSPs are likely to be driven by nuclear cells' intrinsic propensity to fire regular trains of simple spikes. When bathed in tetrodotoxin to block voltage-gated Na channels and prevent firing, cerebellar nuclear cells rest at unusually depolarized potentials, near −40 mV, owing to an apparently low density of leak K conductances relative to a leak-like tonic cation conductance (Raman et al., 2000), probably carried by NALCN1 (Lu et al., 2007). As a consequence, after a perturbation such as a brief hyperpolarization by an IPSP, cells seek a “resting” potential that is above threshold, and, barring further inhibitory input, inevitably produce a spike. This scenario, of Purkinje-activated GABA_A_ receptors generating the primary currents that hold nuclear cells below threshold, presents another apparently ideal specialization for action potentials serving to signal the transient relief of inhibition.

## Synchrony throughout motor pathways

The proposal that synchronous Purkinje activity produces precise spike timing in the cerebellar nuclei raises the question of whether cerebellar inputs or targets also display coherent firing associated with movement. In fact, temporally precise spiking associated with synchronous neuronal firing appears to be a common theme in motor structures. For example, in the primary motor cortex (M1) of behaving monkeys, spike synchronization is observed during voluntary movement, even without significant changes in firing rates, giving rise to the notion that dynamically organized cell assemblies are involved in generating movement (Riehle et al., 1997; Baker et al., 2001). Further supporting this idea, M1 neurons in macaques have been shown to increase their synchrony upon initiation of movement, and analysis of the M1 spike trains reveals that synchronous activity encodes more information about a movement than the mean firing rate alone (Hatsopoulos et al., 1998). Additionally, synchrony increases with training in monkeys, leading to the proposal that synapses that are active during a successful task are reinforced, thereby recruiting increasing numbers of cortical neurons into a synchronous population (Schieber, 2002; Kilavik et al., 2009). Correlations between MEG and EMG signals in people performing motor tasks have been interpreted to indicate that synchronously active neurons in M1 may preferentially recruit motor neurons and muscle fibers in humans, as well (Schoffelen et al., 2005). Even in some sensory systems, although synchronous events can be sparse, sampling over populations of cortical neurons reveals a “synchrony code” that can encode information about somatosensory stimuli (Jadhav et al., 2009), although the significance of such coding strategies remains debated (Shadlen and Movshon, 1999).

A recurring theme in motor systems, however, is that precisely timed spiking occurs in phase with broader oscillatory neural activity. For instance, a recent report showed that M1 neurons that fire coherently with local field potential (LFP) beta frequency (10–15 Hz) oscillations selectively predict motor performance in a coordinated arm and eye movement task in monkeys, leading to the suggestion that LFP oscillations help coordinate activity in distant, distinct cortical areas that control arm reaching and saccadic eye movements (Dean et al., 2012). The source of cortical oscillations is not known, but one possibility is that the cerebellum may help produce or support them. Indeed, beta frequency oscillations are observed in the cerebellum (Courtemanche et al., 2002; D'Angelo et al., 2009), are coherent between the cerebellum and the cortex during sustained movements in monkeys (Soteropoulos and Baker, 2006), and may be causally related (Holdefer et al., 2000). In addition, it has been proposed that synchronous Purkinje activity is organized by ultrafast oscillations (~200 Hz) in the cerebellar cortex (de Solages et al., 2008). Interestingly, simultaneously recorded cerebellar nuclear neurons occasionally fire synchronously themselves, though this behavior has been seen only rarely (Soteropoulos and Baker, 2006). Moreover, both Purkinje neuron and nuclear neuron firing is phase-locked to beta band LFPs in the motor cortex during steady muscle contraction (Holdefer et al., 2000; Courtemanche et al., 2002; Courtemanche and Lamarre, 2005). It is possible that synchronous spiking by nuclear neurons is evoked by synchronized Purkinje neurons, and oscillatory activity is then relayed to M1 and to muscle groups. Consistent with the latter idea, nuclear neurons show some beta frequency oscillations that are coherent with EMG oscillations in shoulder and wrist muscles, and single pulse microstimulation in the cerebellar nuclei evokes several cycles of periodic muscle activity (Aumann and Fetz, 2004). The relationship between activity in the cerebellum and cerebral cortex is not necessarily unidirectional, however, and may include cortical oscillations driving cerebellar waves (Rowland and Jaeger, 2008; Roš et al., 2009; Rowland et al., 2010).

## Factors affecting the likelihood of synchrony coding at corticonuclear synapses

Two major aspects of the hypothesis that the degree of Purkinje cell synchrony affects nuclear cell output, which we refer to as “synchrony coding,” are well supported by evidence: first, Purkinje cells indeed synchronize their simple spikes during behaviors, and second, the biophysical specializations of nuclear cells are well suited to permit entrainment to synchronized IPSPs. Nevertheless, to what extent, if at all, and under what conditions, if any, a time-locked response to synchronous IPSPs is a significant mechanism for encoding cerebellar output has yet to be demonstrated. At the level of the cerebellar cortex, specific remaining questions are how many Purkinje cells actually synchronize in response to specific stimuli, and how many of these cells converge onto common targets. At the level of the nuclei, major questions are whether the naturally occurring fractional synchrony is sufficient to engage time-locking, and how concomitant excitation or neuromodulatory input shapes the response to synchronized inhibition.

None of these questions need have unique answers. The likelihood of simple spike synchrony, for example, may depend on the mode of firing by Purkinje cells. Analyzing data from awake and anesthetized rodents, De Schutter and Steuber (2009) noted bouts of Purkinje cells firing with Poisson statistics and bouts of more regular firing. The periods of regular firing exhibited precise synchrony of inter-spike pauses and, by extension, action potentials, whereas periods of Poisson-like firing did not, leading the authors to propose that Purkinje cells have the capacity to alternate between a rate and a temporal code. Modeling studies suggest that drugs that increase the regularity of firing, which is therapeutic for some forms of ataxia (Walter et al., 2006; Alviña and Khodakhah 2010), raise the probability of Purkinje cell synchrony (Glasauer et al., 2011). Glasauer et al. further predict, however, that high synchrony facilitates an entrainment of nuclear cell spikes to Purkinje cell spikes that may actually be maladaptive for computations requiring more linear integration, such as gaze holding in the oculomotor system (c.f. Lisberger and Fuchs, 1978). Thus, it may be necessary to control synchrony either regionally or according to task.

Regarding regional variation, synchrony of Purkinje cell simple spikes indeed appears to differ across cerebellar cortical areas. In crus I and II, synchrony is more difficult to detect (Heck et al., 2007; Bosman et al., 2010; but see Shin and De Schutter, 2006), whereas it is particularly widespread in the paramedian lobule (Heck et al., 2007; Bosman et al., 2010; Wise et al., 2010). Such differences may be significant, given that patterns of convergence of neighboring Purkinje cells may vary by zone (Sugihara et al., 2009). In addition, synaptic excitation of cerebellar nuclear cells may modulate corticonuclear synchrony coding. Mossy fiber input is expected to precede Purkinje input by two synaptic delays, and, with continuous activation, trains of EPSPs and IPSPs may overlap. Excitation may either facilitate or disrupt the ability of nuclear cells to generate well-timed spikes following synchronous IPSCs. If glutamatergic EPSCs are smoothed, e.g., by prolonged NMDA receptor currents (Gauck and Jaeger, 2003; Pugh and Raman, 2006) or mGluR currents (Zhang and Linden, 2006; Zheng and Raman, 2011), the probability of generating precisely timed post-inhibitory spikes is likely to be increased (Person and Raman, 2012). Noisy or brief excitatory inputs, as predicted for AMPAR-mediated EPSCs, however, may reduce the temporal precision of any code depending on post-inhibitory action potentials.

Regarding alternative mechanisms to synchrony coding, recordings from rat cerebellar slices have presented arguments for linear processing in the cerebellar cortex, by illustrating the linearity of input-output relations of several cerebellar synapses, including parallel fiber synapses and inhibitory synapses onto Purkinje cells (Walter and Khodakhah, 2006). These observations, along with modeling studies of corticonuclear synapses, have led to the suggestion that a linear rate code would provide a particularly information-rich coding strategy (Walter and Khodakhah, 2009). Other computational models, however, predict synchrony coding at corticonuclear synapses as an obligate outcome of Purkinje cell intrinsic properties and convergence, with asynchronous IPSCs effectively suppressing spiking and synchronous input permitting or entraining nuclear firing (Kistler and De Zeeuw, 2003; Glasauer et al., 2011). Related studies include experimental data indicating that Golgi cells in the cerebellar cortex can fire in synchrony; when they do so, they are predicted to exert a relatively weak inhibition on their granule cell targets, while desynchronization of Golgi cell firing by mossy fiber excitation is expected to increase the efficacy of inhibition (Vervaeke et al., 2010). Both electrical and chemical synapses may contribute to this synchrony in Golgi cells (Hull and Regehr, 2012) and this interplay has been illustrated by modeling (c.f. Kopell and Ermentrout, 2004). Abstract models also demonstrate that inhibitory efficacy varies with synchrony (Akam and Kullmann, 2010). Furthermore, the influence of GABA_A_ receptor kinetics on synchrony has been shown at other synapses. In the hippocampus, the kinetics of GABA_A_ receptor mediated currents determine in part the rate at which the network can oscillate, such that rapid decay time constants support high frequency oscillations (Wang and Buzsáki, 1996; Bartos et al., 2002). Similarly, experimental and modeling studies reveal well-timed post-inhibitory spikes after brief IPSGs in the medial superior olive (Dodla et al., 2006).

A parallel example of spike timing shaped by inhibition is evident in the basal ganglia output to the thalamus, an anatomical and physiological sister circuit of the corticonuclear pathway. Spontaneously active, GABAergic internal pallidal neurons, like Purkinje neurons, often show overall increases in firing rate during target thalamic neuron activation (Anderson and Horak, 1985; DeLong et al., 1985; Anderson and Turner, 1991; Inase et al., 1996; Turner and Anderson, 1997; but see also Hikosaka and Wurtz, 1983; Deniau and Chevalier, 1985; Kravitz et al., 2010), leading to the hypothesis that concurrently active pallidal neurons mediate lateral inhibition onto thalamic neurons (Nambu et al., 2002). The paradoxical relationship in firing rates persists, however, in simultaneous recordings of synaptically connected pairs of pallidal and thalamic neurons during sensory relay or movement, which verify that coupled pallidal and thalamic neuron increase their overall firing rates in parallel but can show inverse relationships in instantaneous firing rates (Person and Perkel, 2007; Goldberg and Fee, 2012a,b). Interestingly, thalamic neuron activation is due to concurrent excitatory drive to the thalamus, which overcomes inhibition from the basal ganglia (Goldberg and Fee, 2012a,b). As a result, the effective role of the GABAergic output neurons of the basal ganglia becomes to control the spike timing of thalamic relay neurons.

Resolving whether and when synchrony coding is useful or necessary to cerebellar function will depend in part on understanding what nuclear cells communicate to down-stream targets. In the red nucleus, individual interpositus axons ramify parasagittally (Shinoda et al., 1988), with about 50 axons converging onto each target neuron. Electrophysiological studies of this pathway show that cerebellar nuclear-to-red nucleus axons produce EPSPs with little short-term plasticity (Toyama et al., 1970). These properties may, in principle, allow nuclear input to the red nucleus to drive spiking that follows the firing pattern of the synchronized Purkinje subpopulation. Similarly, large EPSPs have been measured in the cerebellar recipient areas of ventrolateral thalamus (Sawyer et al., 1994). Thus, well-timed post-inhibitory spikes in cerebellar nuclear neurons may be a mechanism whereby both the rate and timing of signals from varying groups of synchronized Purkinje cells are preferentially transmitted to premotor areas and other cerebellar targets. Given the wide variety of structures receiving cerebellar nuclear cell input—the red nucleus, thalamic nuclei, inferior olive, and cerebellar cortex, among others—the relevant aspects of cerebellar output signals may not be homogeneous. Instead, the idea that temporal and rate coding coexist in the cerebellum, either alternating or simultaneously, may be central to resolving the ways in which nuclear cells transduce and transform inputs from Purkinje cells.

### Conflict of interest statement

The authors declare that the research was conducted in the absence of any commercial or financial relationships that could be construed as a potential conflict of interest.
